# Enhanced thermoelectric performance in three-dimensional superlattice of topological insulator thin films

**DOI:** 10.1186/1556-276X-7-570

**Published:** 2012-10-16

**Authors:** Zheyong Fan, Jiansen Zheng, Hui-Qiong Wang, Jin-Cheng Zheng

**Affiliations:** 1 Department of Physics, and Institute of Theoretical Physics and Astrophysics, Xiamen University, Xiamen, Fujian, 361005, People’s Republic of China; 2 Fujian Key Laboratory of Semiconductor Materials and Applications, Xiamen University, Xiamen, Fujian, 361005, People’s Republic of China; 3 Fujian Provincial Key Laboratory of Theoretical and Computational Chemistry, Xiamen University, Xiamen, Fujian, 361005, People’s Republic of China

## Abstract

We show that certain three-dimensional (3D) superlattice nanostructure based on Bi_2_Te_3_ topological insulator thin films has better thermoelectric performance than two-dimensional (2D) thin films. The 3D superlattice shows a predicted peak value of *ZT* of approximately 6 for gapped surface states at room temperature and retains a high figure of merit *ZT* of approximately 2.5 for gapless surface states. In contrast, 2D thin films with gapless surface states show no advantage over bulk Bi_2_Te_3_. The enhancement of the thermoelectric performance originates from a combination of the reduction of lattice thermal conductivity by phonon-interface scattering, the high mobility of the topologically protected surface states, the enhancement of Seebeck coefficient, and the reduction of electron thermal conductivity by energy filtering. Our study shows that the nanostructure design of topological insulators provides a possible new way of *ZT* enhancement.

## Background

The search of good thermoelectrics with high figure of merit [1,2]

(1) $$ZT=\frac{S^{2}\sigma T}{\kappa_{e}+\kappa_{l}}$$

is usually baffled by the competition of the Seebeck coefficient *S*, the electrical conductivity *σ*, the electron thermal conductivity *κ*_e_ and the lattice thermal conductivity *κ*_l_. Recent discoveries that some of the best thermoelectric materials such as Bi_2_Te_3_[1] are also strong 3D topological insulators [3-5], and experimental studies of the mechanical exfoliation and growth of quintuple layers (QL, 1 QL ≈ 0.748 nm) of Bi_2_Te_3_[6,7] attract much interest [8-11] in the thermoelectric properties of thin films of Bi_2_Te_3_ with one or a few QL.

High *ZT* values of Bi_2_Te_3_ thin films depend crucially on the opening of a subgap at the surface, which disappears quickly with the increasing of the film thickness, as suggested both theoretically [12-14] and experimentally [15,16]. Relatively accurate density functional theory calculations [16,17] show that the (indirect) surface gap of Bi_2_Te_3_ vanishes as soon as the thickness of the thin film increases to 3QL. Despite the high mobility [18] of the surface electrons, the gapless surface states would lead to poor thermoelectric performance due to low Seebeck coefficient and high electron thermal conductivity. However, by creating suitable nanostructures, extra energy-dependent electron scattering mechanisms can be introduced, which could increase the Seebeck coefficient [19,20] and reduce the electron thermal conductivity. The consideration of nanostructures of thin films is also motivated by the fact that a single layer of thin film is not of much practical use for thermoelectric applications, and stacks of thin films have much lower lattice thermal conductivity compared with the bulk [21].

**Figure 1:**
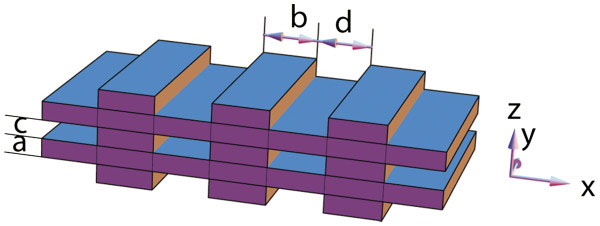
**A sketch of the physical system to be studied.** The system can be regarded as a 3D superlattice with one thin film (thickness *a*) and one stripe-shaped layer (thickness *c* along the *z* direction and width *b* along the *x* direction) stacked alternatively; the spacing between two adjacent stripes is *d*. The transport direction is along the *x* axis.

In this paper, we propose a 3D superlattice nanostructure based on thin films of Bi_2_Te_3_ topological insulator, with one thin film (thickness *a*) and one stripe-shaped layer (thickness *c* along the *z* direction and width *b* along the *x* direction) stacked alternatively; the spacing between two nearby stripes is *d*, as shown in Figure 1. The system is considered to be infinite along the *y* direction and periodic in the *x* and *z* directions. We are interested in thermoelectric transport along the *x* direction. Along the transport direction, the surface carriers will encounter potential barriers/wells if the surface gap is different from the bulk gap. We will give a comparison study of the thermoelectric properties of 2D thin films and the 3D superlattice and show that the latter has higher *ZT* values due to the enhanced Seebeck coefficient and reduced electron thermal conductivity through energy filtering as well as the reduced lattice thermal conductivity by interface phonon scattering. More importantly, the 3D superlattice shows high *ZT* values even when the surface states are gapless, in which case 2D thin films have low *ZT* values.

The 3D superlattice structure proposed here can be regarded as bulk Bi_2_Te_3_ which is nanoporous and resembles nanoporous Si [22] and nanoporous Ge [23], both of which show significant enhancement of the figure of merit due to orders of magnitude reduction in the lattice thermal conductivity. The difference is that the electronic transports for nanoporous Bi_2_Te_3_ and Si/Ge are dominated by surface and bulk carriers, respectively.

## Methods

**Figure 2:**
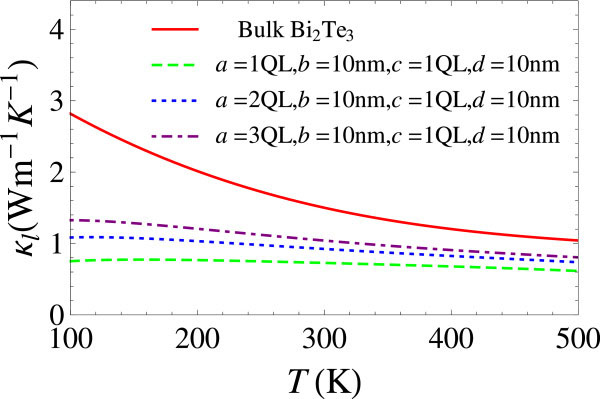
**Lattice thermal conductivities.** Lattice thermal conductivities for bulk Bi_2_Te_3_ obtained by fitting of experimental data [26,27] and for the 3D superlattice with different geometric parameters calculated by Equation 2.

We first consider the lattice thermal conductivity which depends on the geometric parameters and can be estimated using the modified effective medium approximation [24] which treats the system as a nanocomposite. In our case, we can perceive the system as a nanocomposite with vacuum (hole) included in the bulk phase. Taking the thermal conductivity of the holes to be zero, we can express the effective lattice thermal conductivity of the 3D superlattice along the *x* direction as

(2) $$\kappa_{l}=\frac{{\text{κ}}_{\text{b}}}{\Lambda_{b}}\frac{1}{1/\Lambda_{b}+1/\Lambda_{c}}\frac{1-\phi }{1+\phi /2}$$

where *K*_b_ and *Λ*_b_ are lattice thermal conductivity and phonon mean free path (MFP) for the bulk phase, respectively; $\Lambda_{c}=\frac{\left(a+c\right)\left(b+d\right)}{c}$ is the MFP corresponding to the collision of the phonons onto the holes, and $\phi =\frac{cd}{\left(a+c\right)\left(b+d\right)}$ is the volume fraction of the holes. The derivation of *Λ*_b_ proceeds as follows: The effective area of collision for a phonon upon a rectangular vacancy is *cΔy*, where *Δy* is an arbitrary length in the *y* direction; if a phonon travels at a distance *L*, it will encounter *N* = *cΔyLn* vacancies, where $n=\frac{1}{\left(a+c\right)\left(b+d\right)\text{Δ}y}$ is the number density of the vacancies. The MFP *Λ*_c_ is thus $\frac{L}{N}=\frac{\left(a+c\right)\left(b+d\right)}{c}$. The well-defined values for the bulk phase phonon MFP can be extracted [25] from experimental values of the bulk lattice thermal conductivity [26,27] and phonon dispersions. Figure 2 exhibits significant reduction of the lattice thermal conductivity of the 3D superlattice from that of bulk Bi_2_Te_3_. Note that the effective lattice thermal conductivity for the 3D superlattice is weakly temperature-dependent, indicating the dominance of interface scattering. Our result is qualitatively consistent with those from molecular dynamics simulations [9] and experimental measurements [21] on similar nanostructures. Generally, Equation 2 overestimates the lattice thermal conductivity since it ignores other possible phonon scattering channels. As for the lattice thermal conductivity of a single thin film, we take it to be the same as the bulk value, according to molecular dynamics simulation results [9].

A comparison with nanoporous Si [22] and Ge [23] is helpful. While the values of lattice thermal conductivities for bulk Bi_2_Te_3_, Ge and Si range from a few watts per meter Kelvin to several hundred watts per meter Kelvin, the values for the corresponding nanoporous materials are all reduced to below 1 W/(m K). This fact is another indication of the dominance of phonon-interface scattering over the phonon-phonon scattering.

We then consider electron transport coefficients. Since the system is considered to be infinite, the electronic transport is diffusive and Boltzmann's formalism applies. By solving the Boltzmann transport equations with the relaxation time approximation, one can express the thermoelectric transport coefficients as [28]

(3) $$\sigma =e^{2}X_{0}$$

(4) $$S=\frac{-1}{eT}\left(\frac{X_{1}}{X_{0}}-\mu \right)$$

(5) $$\kappa_{e}=\frac{1}{T}\left(X_{2}-\frac{X_{1}^{2}}{X_{0}}\right)$$

where *e* is the magnitude of the elementary charge, *T* is the absolute temperature, *μ* is the chemical potential, and the integrals (*f* is the Fermi-Dirac distribution)

(6) $$X_{n}=\overset{\infty }{\underset{-\infty }{\int }}\left(-\frac{\partial f}{\partial E}\right)\text{Σ}\left(E\right)E^{n}\text{d}E\;\left(n=0,1,2\right)$$

are functionals of the transport distribution function (TDF) [29,30]

(7) $$\Sigma \left(E\right)=\Sigma_{k}v_{x}{\left(k\right)}^{2}\tau \left(k\right)\delta \left(E-E\left(k\right)\right)$$

which are determined by the electronic structure and the electron scattering mechanisms of the material. For simple band structure, the TDF is a product of density of states *g*(*E*), velocity square along the transport direction *v*_*x*_(*E*)^2^, and electron relaxation time *τ*(*E*):

(8) $$\Sigma \left(E\right)=g\left(E\right)v_{x}{\left(E\right)}^{2}\tau \left(E\right)$$

The electronic structures of thin films of topological insulator differ significantly from that of the bulk. First, as the thickness of the film is reduced to one or a few QL, the spin-polarized surface states at one surface will mix up with the components of the opposite spin from the other surface and lead to a hybridization gap at the Dirac point to avoid the crossing of bands with the same quantum number [12-17]. Second, at this thickness, the layers underneath the film surface should be treated as quantum well, which is indicated by experimental observations [15]. In this paper, we adopt a simple parameterization [8] of the dispersion relation of the surface states

(9) $$E\left(k\right)=\pm \sqrt{{\left(V_{\text{D}}\hbar k\right)}^{2}+{\text{Δ}}_{f}^{2}}$$

where ‘±’denotes the conduction (valance) band, *V*_D_ of approximately 4 × 10^5^ m/s [4,18] is the Dirac velocity, and 2 ∆_*f*_ is the surface gap. The zero of energy is chosen to be at the center of the surface gap. This simple dispersion relation is derived from symmetry arguments [8] and exhibits the essentials of thermoelectric properties of the surface states. From the above dispersion relation, we can express the velocity square along the transport direction and the effective 3D density of states for a thin film with thickness *a* as

(10) $$v_{x}{\left(E\right)}^{2}=\frac{\left(E^{2}-{\text{Δ}}_{f}^{2}\right)V_{D}^{2}}{2E^{2}}$$

(11) $$g\left(E\right)=\frac{E}{\pi V_{D}^{2}\hbar^{2}a}$$

For the 3D superlattice structure, the square of velocity along the transport direction takes the same form as in Equation 10, and the effective 3D density of states is that of Equation 11 as scaled by *a*/(*a* + *c*). Since the quantum well states lie much above, we can safely disregard them and consider the surface states only. We also only consider the conduction band with *E* > 0.

To compute the electronic transport coefficients, we should also find an estimation of the electron relaxation time. Experimental studies [18] show that the surface electron mobility *μ*_s_ of Bi_2_Te_3_ approaches 10^4^ cm^2^ V^−1^ s^−1^, about an order of magnitude higher than the bulk value. We use this experimental value of mobility to calculate the intrinsic surface electron relaxation time *τ*_s_. For the superlattice, the surface carriers also encounter potential barriers/wells whenever they reach the boundaries of the surfaces (located at the crossing lines between the thin films and the strips) and suffer from additional scattering. The strips are modeled by rectangular potential barriers/wells with height *V*_i_ = *Δ*_*b*_ − *Δ*_*f*_ and width *b* and average distance *L* = *b* + *d*. Let the transmission probability for the charge carriers with energy *E* through a single strip be *P*(*E*). The path length after passing through the *n*-th strip and scattering by the (*n* + 1)-th one is *nLP*(*E*)^*n*^(1 − *P*(*E*)). The mean free path is the sum of all of the possible path lengths [31], ∑ _*n* = 1_^*∞*^*nLP*(*E*)^*n*^(1 − *P*(*E*)) = *LP*(*E*)/(1 − *P*(*E*)). The corresponding additional relaxation time τa is the mean free path divided by the velocity,

(12) $$\tau_{\text{a}}=\frac{b+d}{v_{x}\left(E\right)}\frac{P\left(E\right)}{1-P\left(E\right)}$$

The transmission probability *P*(*E*) is determined by the interface potential barrier/well according to the following standard quantum mechanical calculations:

(13) $$P\left(E\right)=\frac{1}{1+\frac{{\left(\Delta_{b}-\Delta_{f}\right)}^{2}}{4\left(E-\Delta_{f}\right)\left(E-\Delta_{b}\right)}sin^{2}\left(\sqrt{\frac{2m^{*}\left(E-\Delta_{b}\right)}{\hbar^{2}}}b\right)}$$

The total surface electron relaxation time *τ* for the superlattice is given by

(14) $$\frac{1}{\tau }=\frac{1}{\tau_{s}}+\frac{1}{\tau_{a}}$$

This method of calculating the total electron relaxation time has been recently applied to the study of thermoelectric properties of nanocomposites [32,33]. We assume 2*Δ*_*b*_ = 0.15 eV for bulk Bi_2_Te_3_ according to the experimental value [34]. The surface gap is chosen to be 2*Δ*_*f*_ = 0.3, 0.06, and 0 eV for thin films with thickness 1QL, 2QL, and 3QL, respectively, as suggested by first-principle calculations [17]. The effective mass m^*^ entering Equation 13 stands for that of bulk Bi_2_Te_3_, which has a highly anisotropic effective mass tensor, with the in-plane components 0.021 *m*_0_ and 0.081 *m*_0_ and the out-of-plane component 0.32 *m*_0_ (*m*_0_ is the mass of a free electron) [35]. For simplicity, we take m^*^ to be 0.1 *m*_0_ in our calculations. The exact value of m^*^ is not very crucial for our discussions, since it only affects the optimal value of *b*.

## Results and discussion

**Figure 3:**
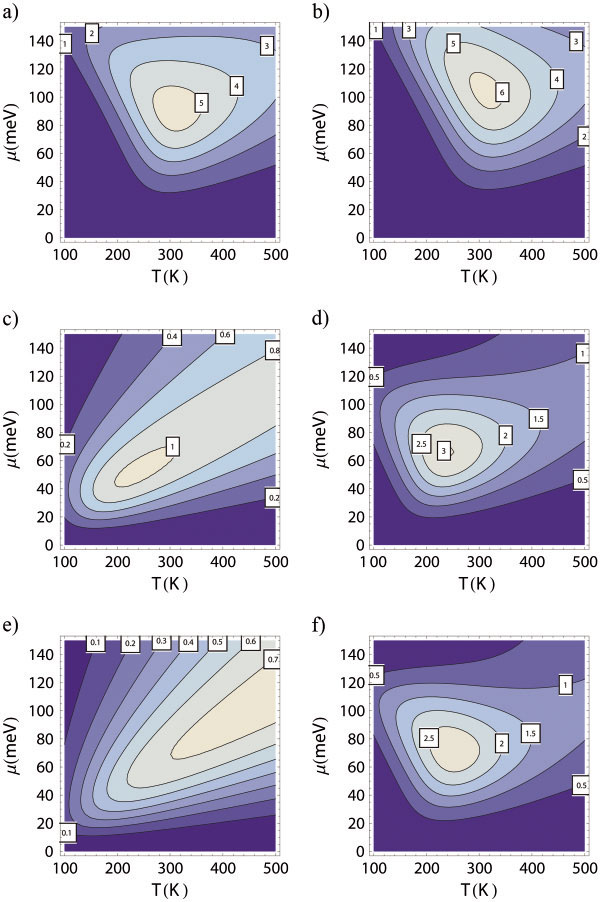
**Figure of merit. ***ZT * values as functions of temperature and chemical potential for Bi_2_Te_3_ 2D thin films with thickness 1QL (**a**), 2QL (**c**), and 3QL (**e**); and the 3D superlattice with *a* = 1QL (**b**), 2QL (**d**) and 3QL (**f**). For the *a* = 1QL case, *c* = 1QL, *b* and *d* = 10 nm; for the *a* = 2QL case, *c* = 2QL, *b* = 7.5 nm and *d* = 10 nm; for the *a* = 3QL case, *c* = 3QL, *b* = 7.5 nm and *d* = 10 nm. The zero of energy is chosen to be at the center of the surface gap.

Figure 3 shows the calculated *ZT* values for 2D thin films and the 3D superlattice with varying geometric parameters. Both of the stand-alone thin film with thickness of 1 QL and the 3D superlattice with *a* = 1QL exhibit high figure of merit *ZT* of 5 to 6 at room temperature with appropriate doping level. For thicker films, the surface gap becomes smaller and yields lower *ZT* values. However, the 3D superlattice is much more robust against the disappearance of surface gap and still shows peak value of *ZT* of approximately 2.5 (Figure 3f) for gapless states. In contrast, a gapless thin film with a thickness of 3QL demonstrates no better performance than the bulk material (Figure 3e).

**Figure 4:**
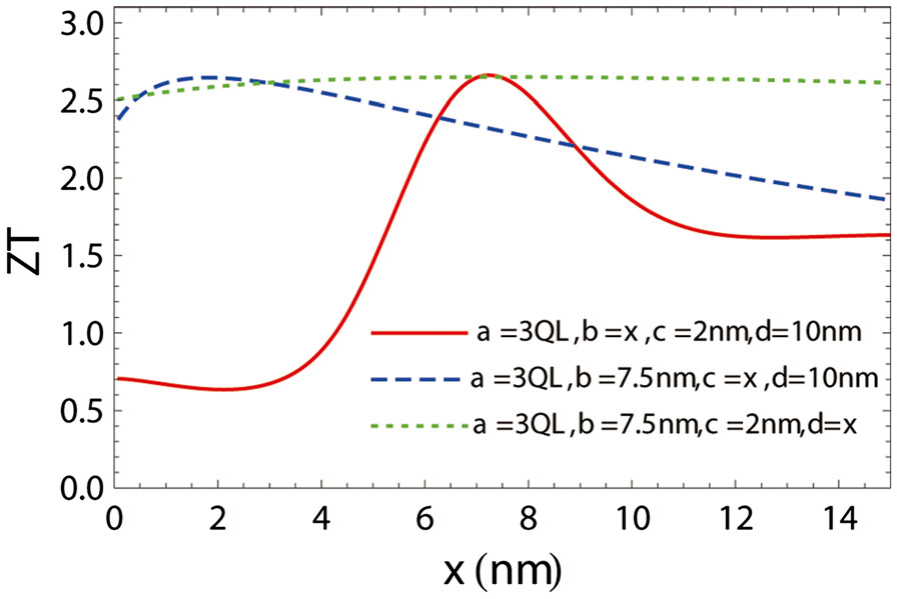
**Optimized geometric parameters. ** Optimization of the geometric parameters *b*, *c*, and *d* for the 3D superlattice with *a* = 3QL at *T* = 250 K and *μ* = 60 meV. The geometric parameter dependence of *ZT* values for 3D superlattice with *a* = 2QL and 1QL has similar trends as given here for the case of *a* = 3QL.

*ZT* values depend on the geometric parameters *a*, *b*, *c* and *d* since both the lattice thermal conductivity and the electronic transport coefficients depend on these parameters. The dependence of *ZT* on the geometric parameters for *a* = 3QL case is shown in Figure 4. While *ZT* has a weak dependence on *d*, it depends strongly on *b* since this parameter largely affects the transmission probability *P*(*E*) and hence the electron relaxation time. Only an appropriate *b* gives the desired energy filtering effect. The weak dependence of *ZT* on *d* results from the fact that the phonon collision probability 1/Λ_c_ and the hole volume fraction ϕ have opposite dependences on *d* and the fact that the total electron relaxation time is not strongly dependent on *d*. The optimized value of *c* results from the optimization of the *B* factor [36] which represents the relative transport strength of electrons over phonons. A large value of *c* gives a lower lattice thermal conductivity and a lower effective 3D density of states, and only an appropriate value of *c* gives an optimized *B* factor. We should note that our model is only valid for appropriate ranges of these geometric parameters. For example, our model cannot be extrapolated to the *c* = 0 case, since in this limiting case, our model treats the system as parallel thin films with vanishing separation rather than the bulk material. The conclusion of this parametric study leads to the following recommendations for the relevant parameters: *c* takes the same value of *a*, *b* takes the value of 5–10 nm, and *d* takes the value of 10–20 nm.

**Figure 5:**
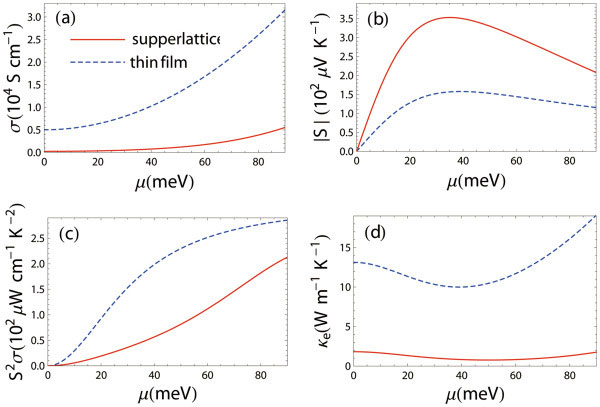
**Thermoelectric transport coefficients.** Thermoelectric transport coefficients as functions of chemical potential at *T* = 250 K for the 2D thin film with thickness 3QL and the 3D superlattice with *a* = 3QL, *b* =7.5 nm, and *c* = 3QL, *d* = 10 nm.

To understand why 3D superlattice outperforms 2D thin films, we plot in Figure 5 the individual thermoelectric transport coefficients as functions of chemical potential at *T* = 250 K for the case of *a* = 3QL, where the difference of the *ZT* values is most significant. As expected, the electrical conductivity of the superlattice is heavily reduced (Figure 5a) due to the additional scattering by the potential barriers/wells. The additional scattering reduces the effective mobility of charge carriers and decreases the electrical conductivity. This reduction of electrical conductivity is not desirable for obtaining high figure of merit. However, this additional scattering also has two beneficial effects on thermoelectric transport, which result in an increase of the Seebeck coefficient and a decrease of the electronic thermal conductivity. Firstly, the additional relaxation time is strongly energy dependent in such a way that charger carriers with energies lower than a certain value are largely scattered back and those with energies higher than a certain value mostly transport through. With an appropriate choice of the barrier/well width *b* and the chemical potential, one can effectively filter out the charge carriers with energies lower than the chemical potential. Since charger carriers with energies above and below the chemical potential contribute oppositely to the Seebeck coefficient (Equation 4), this energy-filtering mechanism can significantly enhance the Seebeck coefficient (Figure 5b). Whether the power factor is increased or decreased due to this energy filtering effect depends on the choices of relevant parameters. In the present case, the power factor is decreased (Figure 5c). From Figure 3e,f, we can infer the optimal chemical potential as about 65 meV. At this value of chemical potential, the power factor for the 2D thin film is about twice as large as that for the 3D superlattice. Then, why does the figure of merit for superlattice reach a value of about 2.5, while that for the thin film only takes a value of about 0.6? This is mainly resulted from the significant reduction of the electronic thermal conductivity for the superlattice compared with the thin film. As can be seen from Figure 5d, the electronic thermal conductivities for the thin film and the superlattice are about 13 and 1 W/(m K) respectively. Combined with the reduction of the lattice thermal conductivity, the total thermal conductivity for the superlattice is about 1/8 of that for the thin film. A combination of all these effects results in a four-fold enhancement of the *ZT* for the superlattice compared with the thin film. The significant reduction of the electronic thermal conductivity also results from the energy filtering mechanism. With the filtering of the low-energy charger carriers, the energy distribution of transport distribution function [29,30] becomes more concentrated, resulting in a violation of the Wiedemann-Franz law [28] which states that the electronic thermal conductivity is proportional to the electrical conductivity, κ_e_ = L_0_σ*T*, with the Lorentz number $L_{0}=\frac{\pi^{2}k_{B}^{2}}{3e^{2}}=2.45\times 10^{-8}$ WΩK^−2^. From another point of view, this violation makes the electronic transport more reversible [37], which is desirable for efficient thermoelectric energy conversion.

To get an intuition of the necessity of violating the Wiedemann-Franz law for superior thermoelectric performance, suppose that the lattice thermal conductivity is reduced to zero, and the Seebeck coefficient is 200 *μ*VK^−1^, then if the Wiedemann-Franz law were strictly valid, we would have achieved a relatively low figure of merit *ZT* = *S*^*2*^/*L*_0_ ≈ 1.6 regardless how large the electrical conductivity would be. Thus, as we approach the lower limit of lattice thermal conductivity, it is imperative to find a way to change the shape of the transport distribution function [29,30] either by altering the electronic structures [38] or by introducing energy-dependent electron scattering mechanisms.

Finally, we add some view points on the approach that we used in this work. For the study of thermoelectric transport of a nanostructured material, there are two complementary ways of viewing the system. One is to take the system as a whole, in which case the nanostructures do alter the electronic structure of the system, but it is difficult to calculate the band structure of such large system directly by first principles method due to large number of atoms presented in nanostructures. Another way is to view the system as some bulk material with nanostructures that do not affect the electronic structure of the bulk material significantly, but introduce some extra scatterings for the charge carriers. We have chosen the second approach in our study. This approach has been widely used in the community of thermoelectrics. For example, in the study of nanocomposites with grain boundaries [32,33], one usually assumes that the electronic structure inside the grain boundary is the same as that of the corresponding bulk material. The grain boundary does not affect significantly the energy-band structure and only serves as a scattering interface. The only difference between our model and the nanocomposite models [32,33] is that our bulk material is quasi-two-dimensional instead of three-dimensional, and the grain boundaries are replaced by the strips in our proposed structure. So long as the average distance between the strips is large compared with the size of the strips (which is the case for the optimized structures), this view point is valid and there is no significant deficiency in our model.

## Conclusions

In summary, we demonstrated that certain nanostructures of topological insulators have the potential of overcoming the obstacle of competition of the individual thermoelectric transport coefficients to achieve high thermoelectric figure of merit. High electron mobility of the topologically protected surface states together with the holy structure of the 3D superlattice ensures a large *B* factor [36], and the energy filtering effect introduced by the inhomogeneous superlattice structure promotes the Seebeck coefficient and the ratio of electrical conductivity to the electron thermal conductivity. The optimal temperature of performance for the 3D superlattice with optimized geometric parameters is around or below room temperature, making it very appealing for thermoelectric power generation and refrigeration applications around and below room temperature, respectively. In addition, a similar structure has appeared in a thin film transistor array, with an insulating substrate and a stripe-shaped semiconductor layer for a plurality of transistors [39], which demonstrates the experimental feasibility of our proposed 3D superlattice structure. The detailed information of geometric and electronic properties of the fabricated superlattice can be characterized by integrated electron scattering and X-ray scattering techniques [40,41].

## Competing interests

The authors declare that they have no competing interests.

## Authors' contributions

ZF carried out the main part of the calculations and drafted the manuscript. JZ carried out part of the calculations. H-QW participated in the organization of the project and discussions of the results and revised the manuscript. J-CZ organized the project, analyzed the results and revised the manuscript. All authors read and approved the final manuscript.
